# From Stroke to the Final Diagnosis: Cardiac Amyloidosis

**DOI:** 10.7759/cureus.113697

**Published:** 2026-07-30

**Authors:** Shahana Alasgarli, Taleh Naghdaliyev, Anar Karimkhanov, Gulay Mammadova

**Affiliations:** 1 Cardiology, Yeni Klinika, Baku, AZE; 2 Nephrology, Yeni Klinika, Baku, AZE

**Keywords:** amyloid deposition, amyloidosis, cardiac amyloidosis, cardioembolic stroke, hcmp, nephrotic syndrome, proteinuria

## Abstract

Cardiac amyloidosis is an infiltrative cardiomyopathy that often presents with nonspecific clinical manifestations, leading to delayed diagnosis. Early recognition is essential, particularly in patients with extracardiac manifestations suggestive of systemic amyloidosis. A 43-year-old woman was admitted with an ischemic stroke of unknown origin. During the evaluation for a potential cardioembolic source, transthoracic and transesophageal echocardiography revealed diffuse myocardial and left atrial abnormalities suggestive of an infiltrative process. Electrocardiography demonstrated low QRS voltage despite increased ventricular wall thickness. Cardiac magnetic resonance imaging showed diffuse late gadolinium enhancement, markedly elevated native T1 values, and increased extracellular volume, strongly supporting the diagnosis of cardiac amyloidosis. Although serum and urine immunofixation and bone marrow biopsy were inconclusive, renal biopsy confirmed monoclonal lambda light-chain deposition, establishing the diagnosis of amyloid light-chain (AL) amyloidosis. The patient received standard heart failure therapy and was referred for daratumumab-based chemotherapy. Despite treatment, she rapidly developed anuric renal failure and multiorgan failure, resulting in death shortly after the second chemotherapy cycle. This case highlights the diagnostic value of multimodality imaging in suspected cardiac amyloidosis and emphasizes the importance of considering systemic amyloidosis in younger patients presenting with stroke, nephrotic syndrome, increased ventricular wall thickness, and low QRS voltage. Early diagnosis remains crucial, although prognosis may be poor in advanced AL cardiac amyloidosis.

## Introduction

Cardiac amyloidosis is an infiltrative cardiomyopathy characterized by the extracellular deposition of misfolded amyloid fibrils within the myocardial interstitium, resulting in progressive ventricular wall thickening, impaired myocardial compliance, and ultimately restrictive cardiomyopathy and heart failure [1]. The disease often presents with nonspecific clinical manifestations, including exertional dyspnea, fatigue, peripheral edema, atrial arrhythmias, conduction abnormalities, and preserved left ventricular ejection fraction in the early stages [1]. These features frequently overlap with more common cardiovascular disorders, such as hypertensive heart disease or hypertrophic cardiomyopathy, leading to substantial diagnostic delays [2].

Early recognition of cardiac amyloidosis is crucial because timely diagnosis enables disease-specific treatment that can slow disease progression, improve quality of life, and enhance survival. Clinicians should maintain a high index of suspicion, particularly in patients presenting with unexplained left ventricular hypertrophy, heart failure with preserved ejection fraction, low-voltage electrocardiographic findings disproportionate to ventricular wall thickness, or extracardiac manifestations suggestive of systemic amyloidosis [3-5]. Such manifestations may include bilateral carpal tunnel syndrome, lumbar spinal stenosis, peripheral or autonomic neuropathy, proteinuria, nephrotic syndrome, macroglossia, periorbital purpura, or unexplained weight loss [6]. A comprehensive diagnostic approach integrating clinical assessment, multimodality cardiac imaging, laboratory evaluation for monoclonal proteins, and tissue biopsy when indicated is essential for establishing an accurate diagnosis and differentiating amyloid subtypes, thereby facilitating appropriate therapeutic management [7].

## Case presentation

A 43-year-old woman was admitted to a tertiary care center with an ischemic stroke of unknown origin. During hospitalization, she underwent a routine cardiology evaluation to identify a potential cardioembolic source of the stroke. Transthoracic echocardiography (TTE) revealed a suspicious finding at the base of the left atrium. Consequently, the patient was referred to our center for further evaluation.

**Figure 1:**
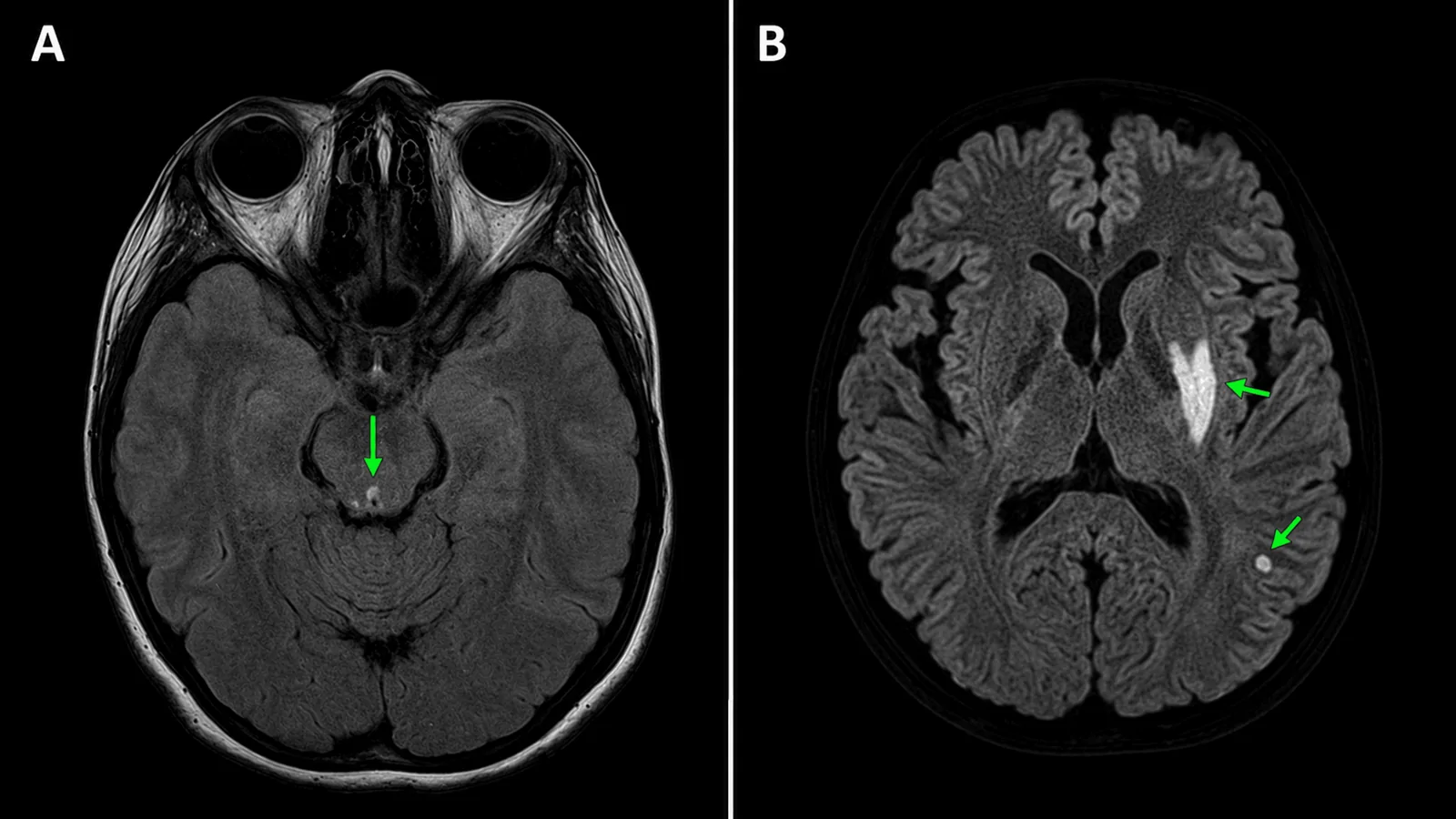
Brain magnetic resonance imaging. A: Axial FLAIR image demonstrating a hyperintense lesion in the pons (arrow). B: Axial diffusion-weighted imaging (DWI) demonstrating multiple acute ischemic infarcts involving the left corona radiata and left temporal lobe (arrow)

At the tertiary care center, the patient underwent brain magnetic resonance imaging (MRI) and a comprehensive stroke evaluation. Brain MRI demonstrated multiple acute ischemic infarcts (Figure 1). No significant carotid or vertebral artery atherosclerotic plaques or occlusive disease were identified, and 48-hour cardiac rhythm monitoring revealed no clinically significant arrhythmias.

On admission, the patient was hemodynamically stable, with a blood pressure of 100/70 mmHg, a heart rate of 80 beats/min, and an oxygen saturation of 94% on room air. Marked bilateral pretibial edema was noted. The patient reported that she had been diagnosed with nephrotic syndrome of unknown etiology six months earlier. Routine laboratory investigations were performed (Table 1).

**Table 1:** Laboratory test results on admission

Lab. test (full name)	Results	Reference value
Hb (hemoglobin)	12.4	12.5–15.1 g/dL
Hct (hematocrit)	36.7	36–47%
PLT (platelet count)	189	184–433 ×10^9/L
WBC (white blood cell count)	15.33	5.2–9.2 ×10^9/L
Creatinine (serum creatinine)	0.63	0.5–0.9 mg/dL
Potassium (K+, serum potassium)	3.57	3.5–5.1 mmol/L
Sodium (Na+, serum sodium)	138	135–145 mmol/L
Urea (serum urea)	53	15–40 mg/dL
BUN (blood urea nitrogen)	25	8–20 mg/dL
NT-proBNP (N-terminal pro-B-type natriuretic peptide)	12.968	<125 pg/mL
TSH (thyroid-stimulating hormone)	7.16	0.27–4.2 mIU/L
CRP (C-reactive protein)	21	<5 mg/L
AST (aspartate aminotransferase)	106	10–35 U/L
ALT (alanine aminotransferase)	55	7–35 U/L
CK-MB (creatine kinase–myocardial band)	41	<25 U/L
Albumin (serum albumin)	3.69	3.5–5.2 g/dL
Urine total protein (spot)	2856	1–14 mg/dL
Total protein/creatinine ratio (UPCR)	21	<0.2

**Figure 2:**
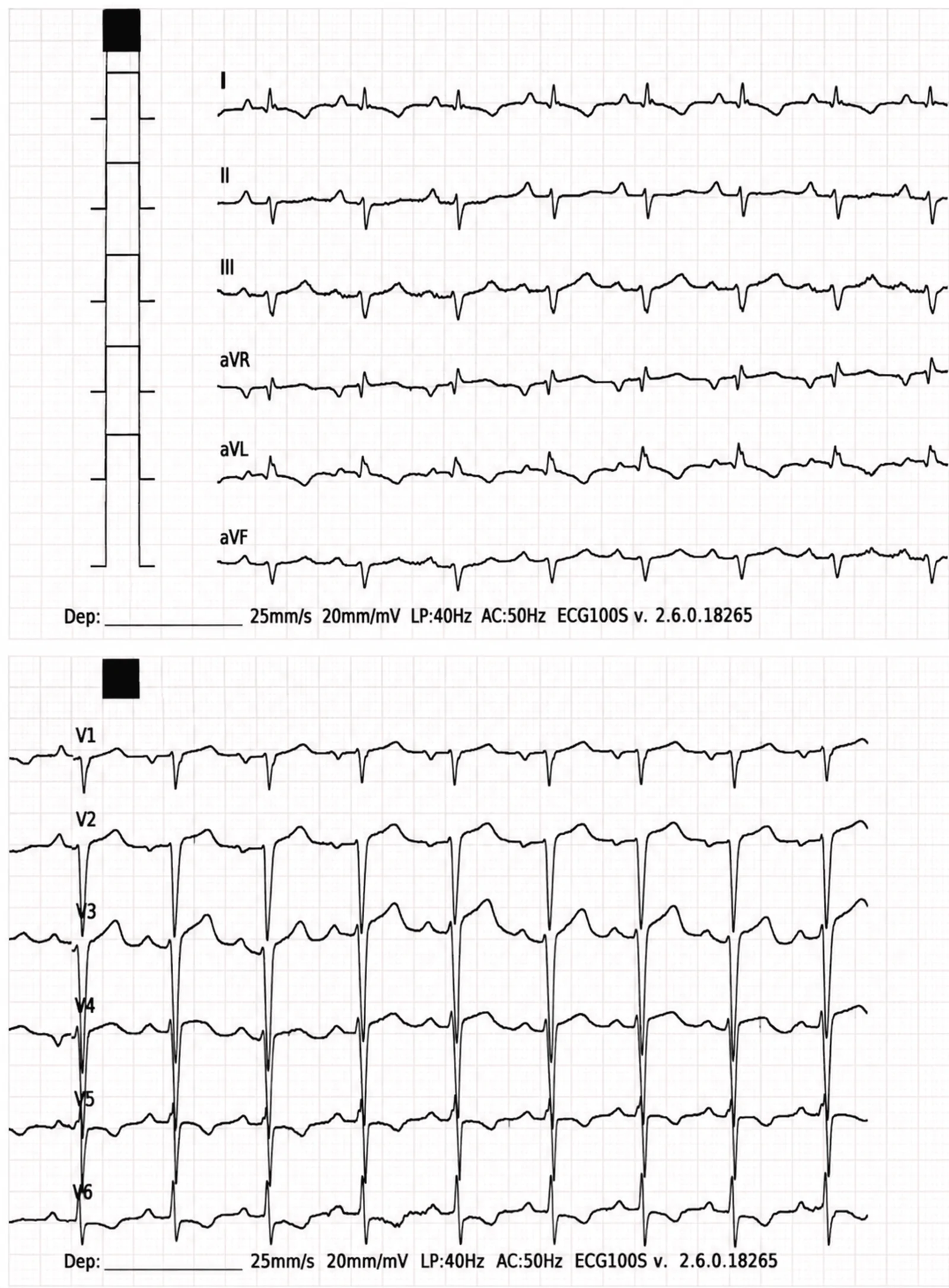
12-lead electrocardiogram on admission. 12-lead electrocardiogram showing sinus rhythm, left axis deviation, poor R-wave progression, and diffuse ST-T abnormalities

A 12-lead electrocardiogram demonstrated sinus rhythm with low QRS voltage (Figure 2). 

**Figure 3:**
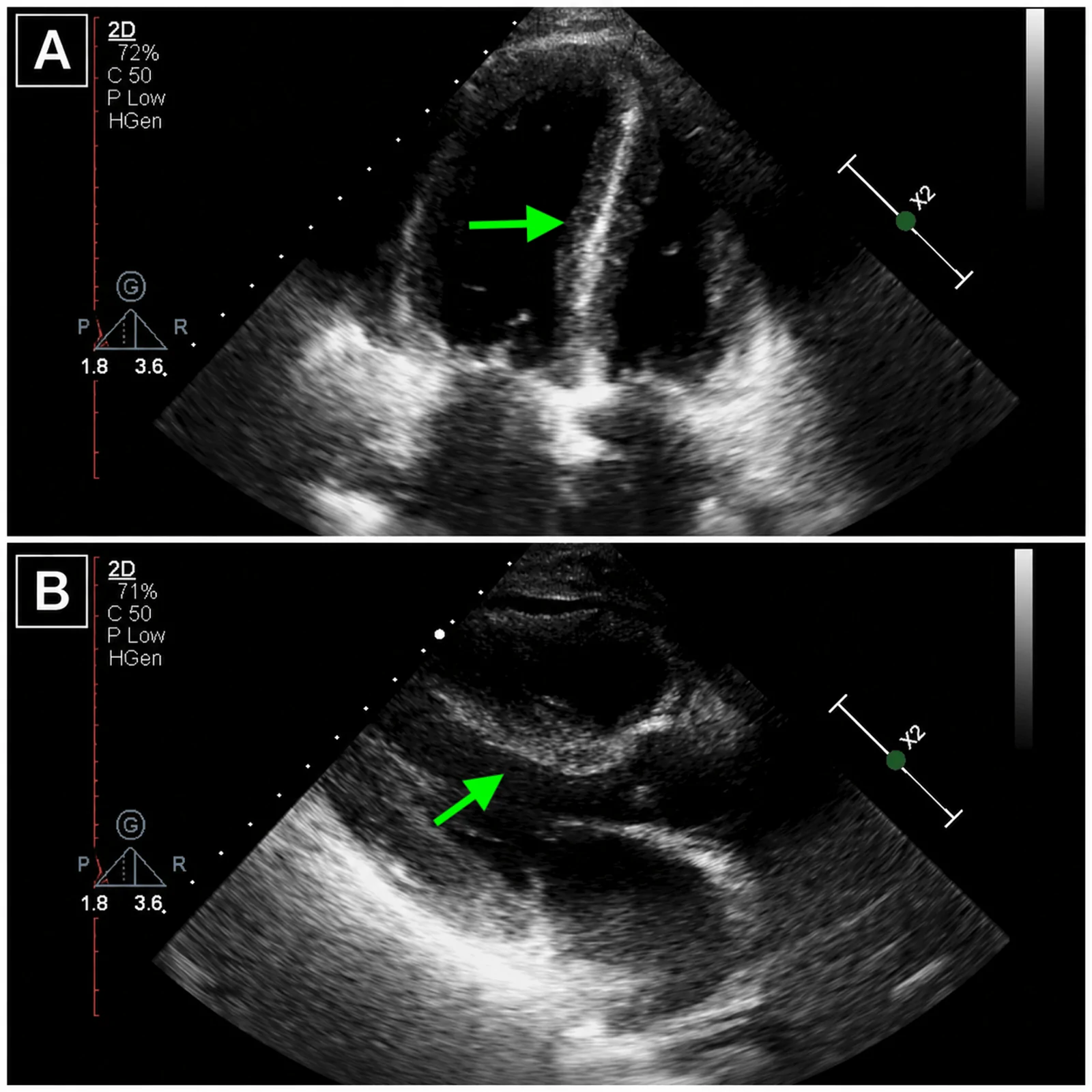
Transthoracic echocardiogram. A: Apical four-chamber view, demonstrating left ventricular hypertrophy (arrow). B: Parasternal long-axis view, also demonstrating left ventricular hypertrophy (arrow)

Comprehensive TTE showed mildly reduced left ventricular systolic function (estimated left ventricular ejection fraction, 40%), increased left and right ventricular wall thickness, interventricular septal hypertrophy, and thickening of the interatrial septum (Figure 3). 

**Figure 4:**
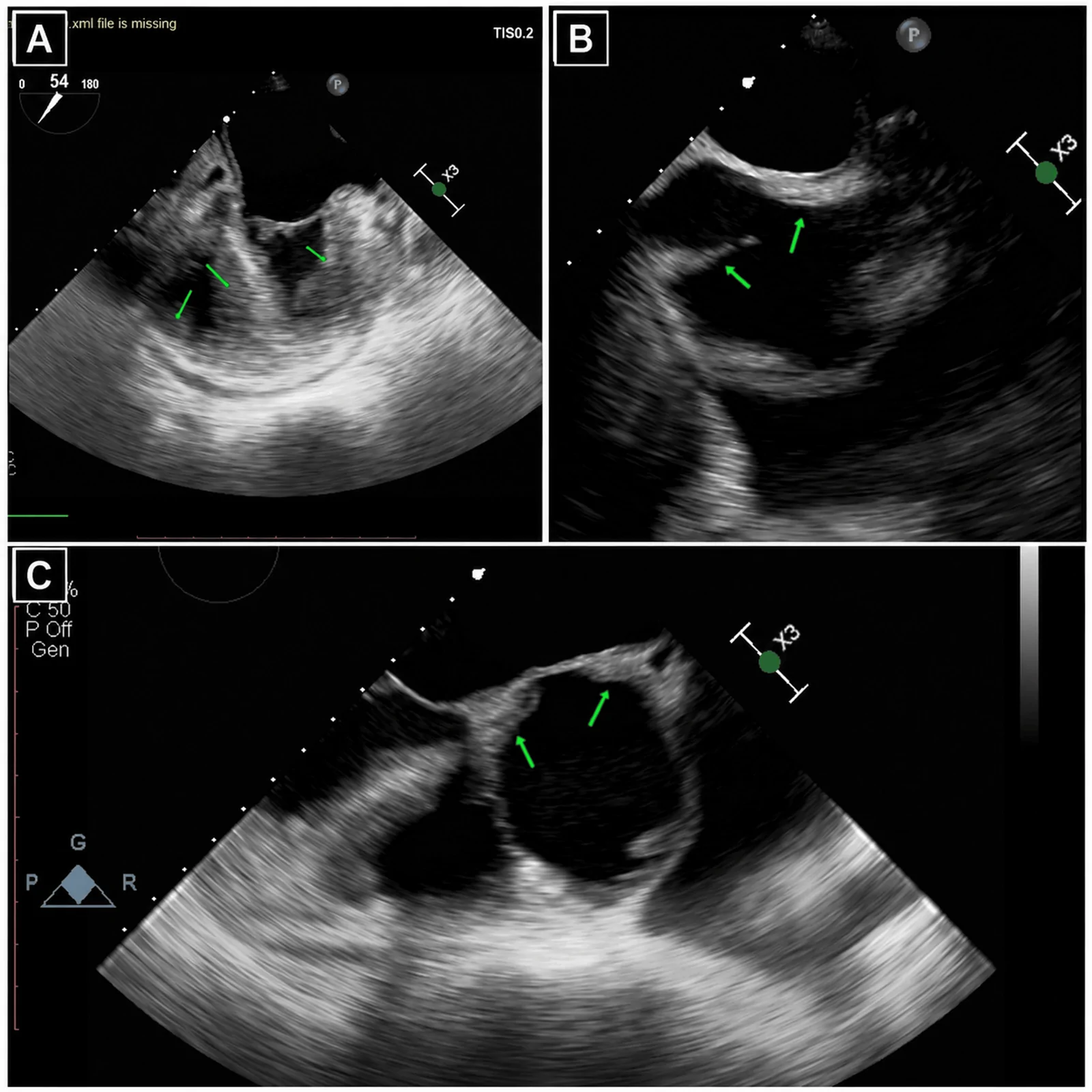
Transesophageal echocardiogram. A: Four-chamber view demonstrating biventricular hypertrophy. B: Bicaval view demonstrating left atrial wall thickening suggestive of infiltrative involvement. C: Four-chamber view also demonstrating left atrial wall thickening suggestive of infiltrative involvement

To further characterize these findings, transesophageal echocardiography (TEE) was performed. TEE demonstrated abnormal infiltrative deposits along the walls of the left atrium (Figure 4).

**Figure 5:**
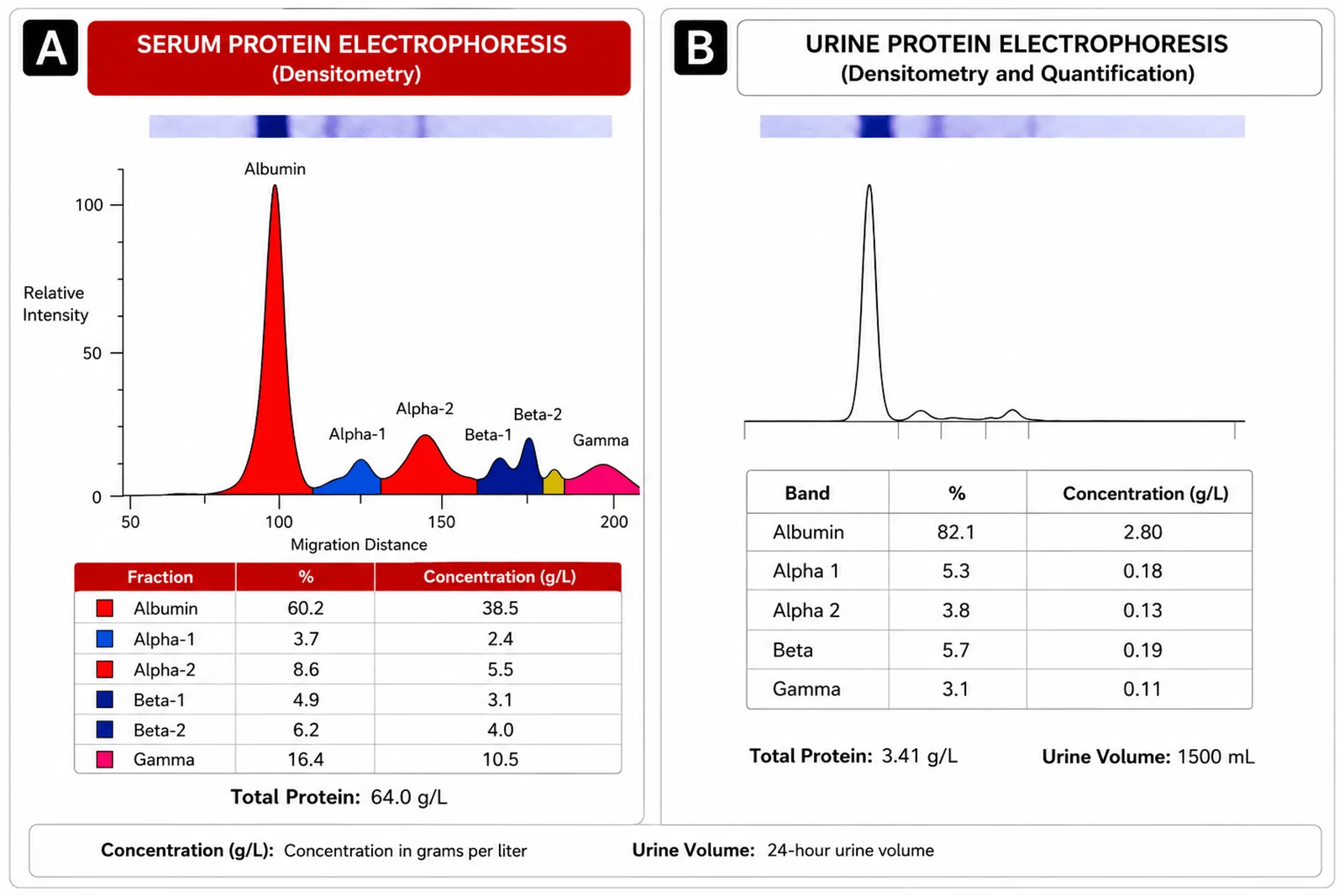
Serum and urine electrophoresis. A: Serum protein electrophoresis demonstrating a normal distribution of protein fractions, including albumin, α1-, α2-, β1-, β2-, and γ-globulin peaks. B: Urine protein electrophoresis (densitometry and quantification) demonstrating predominantly albuminuria without a detectable monoclonal protein (M-spike)

Based on these findings, cardiac magnetic resonance imaging (CMR), serum and urine protein electrophoresis with immunofixation, and bone marrow biopsy were performed. The results are summarized in Table 2 and Figure 5.

**Table 2:** Serum immunoglobulin, total light chain and 24-hour urine protein analysis

Parameter	Serum (Blood)	Urine (24-hour)	Reference Range
IgG	3.78 g/L ↓	N/A	6.50–16.00 g/L
IgA	0.96 g/L	N/A	0.45–3.80 g/L
IgM	3.32 g/L ↑	N/A	0.50–3.00 g/L
Total Kappa light chain	1330 mg/L ↓	91.90 mg/L ↑	Serum: 1700–3700 mg/L; Urine: 0–15 mg/L
Total Lambda light chain	813.0 mg/L ↓	95.20 mg/L ↑	Serum: 900–2100 mg/L; Urine: 0–15 mg/L
Kappa/Lambda ratio	1.64	0.97	Serum: 1.35–2.65; Urine: 0.75–4.50
Total protein	N/A	16.40 g/L ↑	0–0.14 g/L
24-hour urinary protein excretion	N/A	8.20 g/day ↑	0–0.15 g/day

**Figure 6:**
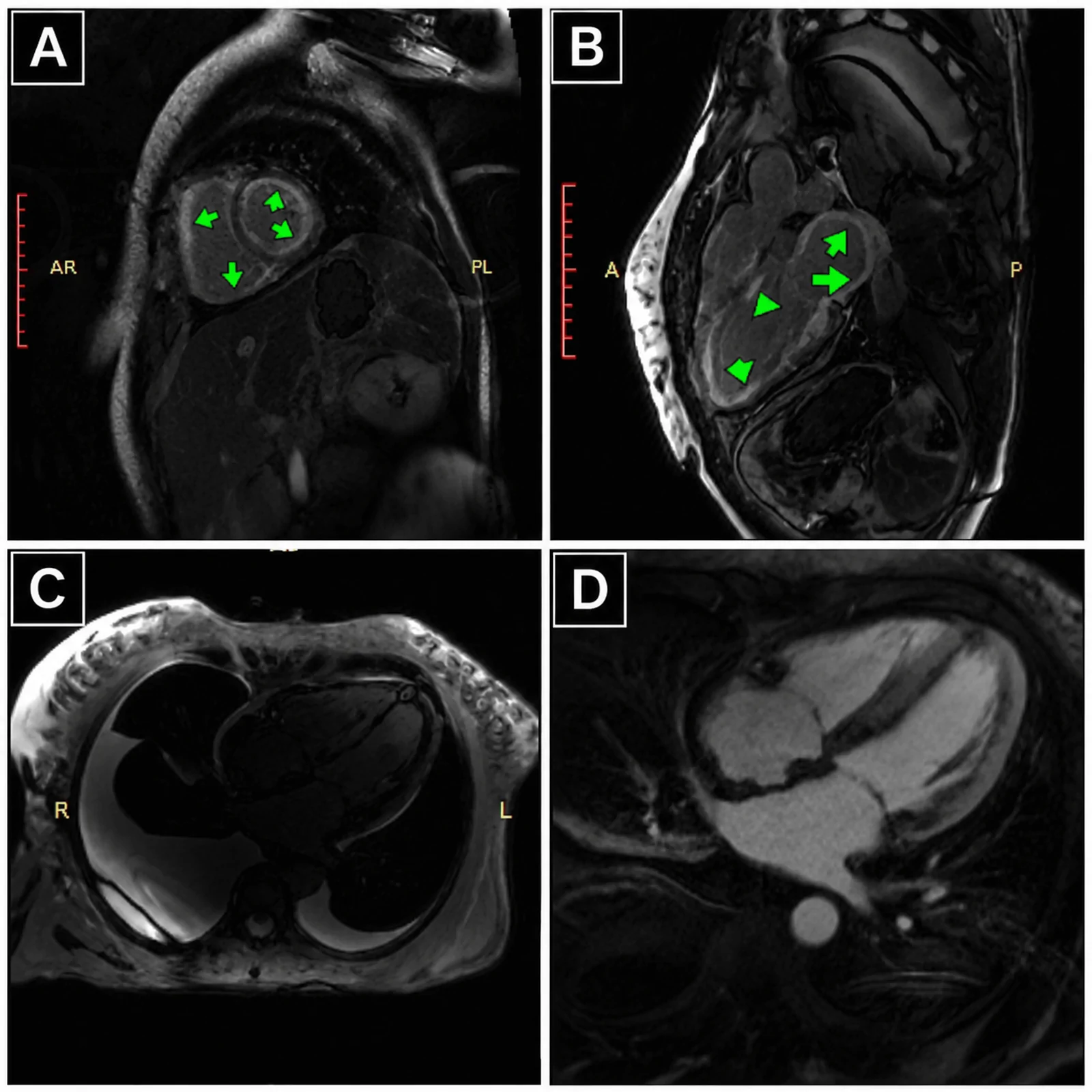
Cardiac magnetic resonance imaging. A: Short-axis view demonstrating diffuse subendocardial LGE in both ventricles (arrows). B: Three-chamber view demonstrating also diffuse subendocardial LGE in the left ventricle and left atrium. C and D: Four-chamber views demonstrating biventricular hypertrophy LGE: Late gadolinium enhancement

Serum and urine immunofixation studies, protein electrophoresis, and bone marrow biopsy were inconclusive. However, CMR demonstrated findings highly suggestive of cardiac amyloidosis (Figure 6).

Native myocardial T1 mapping values were diffusely elevated throughout the left ventricular myocardium, ranging from 1576 to 1676 ms (normal range at 3T: 1211-1333 ms). The synthetic extracellular volume (ECV) was markedly increased at approximately 61% (normal range: 20-26%). Ten minutes after intravenous gadolinium administration, phase-sensitive inversion recovery sequences demonstrated diffuse subendocardial late gadolinium enhancement (LGE) involving the left ventricular walls, patchy mid-myocardial enhancement, subepicardial enhancement within the basal and mid interventricular septum, and focal near-transmural LGE in the basal inferior and basal inferolateral segments, consistent with a mixed non-ischemic LGE pattern. Diffuse LGE was also observed in the walls of the left atrium, right atrium, and right ventricle.

**Figure 7:**
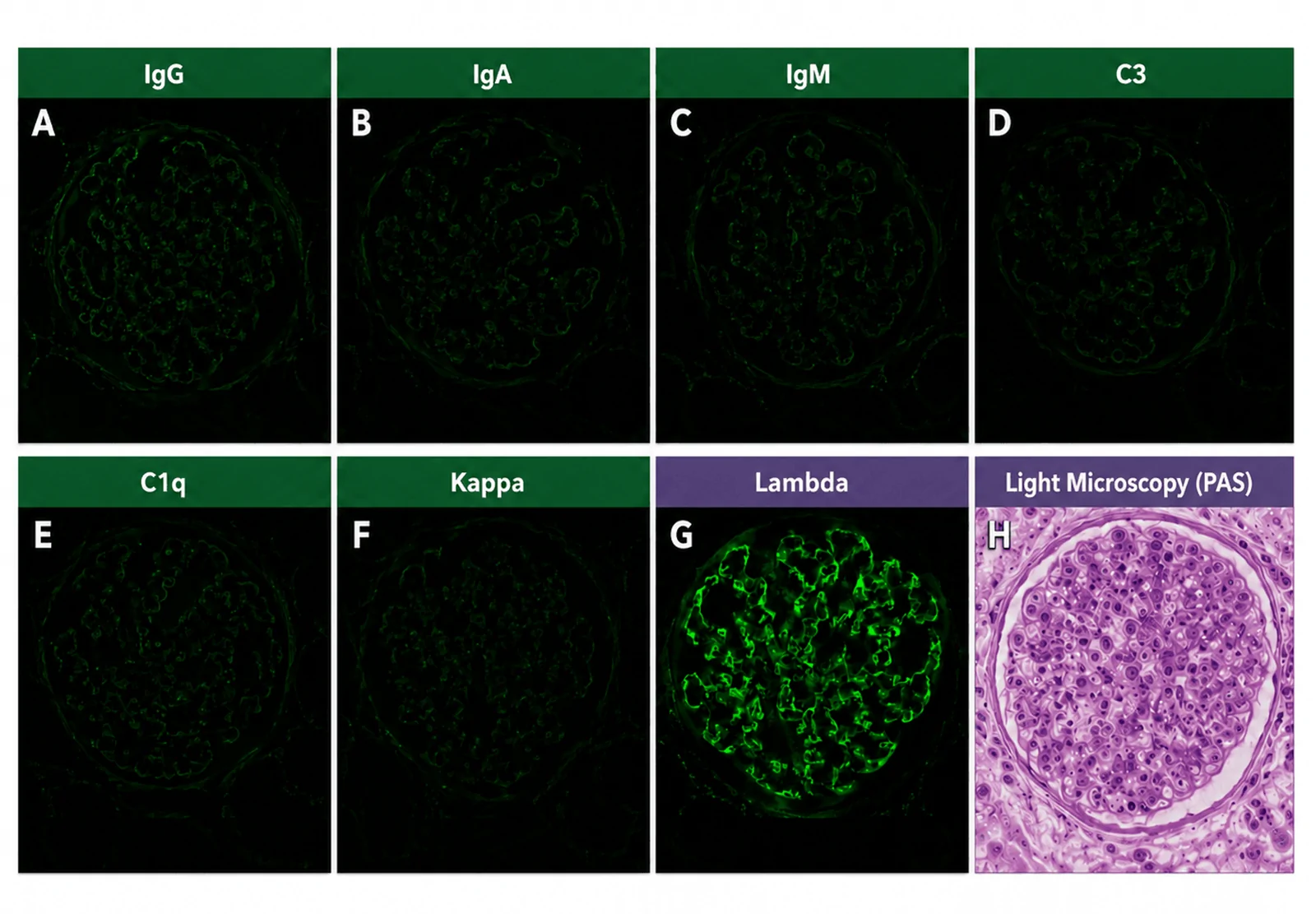
A: Immunofluorescence staining for IgG shows no significant glomerular deposition. B: Immunofluorescence staining for IgA is negative. C: Immunofluorescence staining for IgM is negative. D: Immunofluorescence staining for C3 is negative. E: Immunofluorescence staining for C1q is negative. F: Immunofluorescence staining for kappa light chains is negative. G: Immunofluorescence staining demonstrates strong glomerular positivity for lambda light chains, indicating lambda light chain–restricted deposition. H: Light microscopy with Periodic acid–Schiff (PAS) stain demonstrates glomerular involvement. Immunofluorescence images were obtained at ×40 magnification; PAS-stained light microscopy was examined at ×40 magnification

To establish the diagnosis, a renal biopsy was performed. Renal biopsy specimens were stained with Congo red, which demonstrated characteristic apple-green birefringence under polarized light, confirming the presence of amyloid. Furthermore, immunofluorescence showed strong lambda light-chain positivity within the amyloid deposits, while staining for kappa, IgG, IgA, IgM, C3, and C1q was negative. These findings established the diagnosis of lambda-associated amyloid light-chain (AL) amyloidosis (Figure 7).

Based on the clinical presentation and the results of multimodality imaging and histopathological evaluation, the patient was diagnosed with AL cardiac amyloidosis associated with monoclonal lambda light-chain deposition.

During hospitalization, the patient received apixaban 5 mg twice daily, metoprolol 12.5 mg twice daily, and dapagliflozin 10 mg once daily. Anticoagulation with apixaban was initiated because the patient presented with multiple small ischemic infarcts on brain MRI, a pattern highly suggestive of an embolic mechanism. Comprehensive evaluation did not identify an alternative cause of stroke: no significant carotid or vertebral artery atherosclerosis was detected, no left atrial or left atrial appendage thrombus was identified on transesophageal echocardiography, and 48-hour rhythm monitoring did not reveal atrial fibrillation or other clinically significant arrhythmias. Although the patient remained in sinus rhythm, the stroke was considered most likely to be cardioembolic in origin after exclusion of other potential etiologies. Therefore, apixaban was prescribed for presumed cardioembolic stroke as secondary stroke prevention. She was subsequently referred to a specialized hematology center for chemotherapy and received a regimen consisting of daratumumab, cyclophosphamide, bortezomib, and dexamethasone (Dara-CyBorD).

Following the second cycle of chemotherapy, the patient developed progressive anuria and rapidly worsening renal failure. She required endotracheal intubation and continuous ultrafiltration because of multiorgan dysfunction. Despite intensive supportive care, her clinical condition continued to deteriorate, and she died on the fourth day in the intensive care unit due to multiorgan failure.

## Discussion

Cardiac amyloidosis is an infiltrative cardiomyopathy characterized by the extracellular deposition of misfolded amyloid fibrils within the myocardium, resulting in progressive ventricular stiffness, heart failure, arrhythmias, and thromboembolic complications [1]. AL amyloidosis, caused by monoclonal immunoglobulin light-chain deposition, is the most aggressive form of systemic amyloidosis and is associated with a poor prognosis when cardiac involvement is present [1,2].

Our patient presented with an ischemic stroke of unknown origin, which ultimately proved to be the first manifestation of systemic AL amyloidosis. Brain MRI demonstrated multiple small ischemic infarcts, a pattern that is highly suggestive of an embolic etiology. The patient’s relatively young age and increased left atrial wall thickness further raised the suspicion of a cardioembolic source. Despite the fact that the patient had presented with peripheral edema approximately six months before admission and had been diagnosed with nephrotic syndrome, no comprehensive diagnostic evaluation was performed at that time, and the patient did not attend further medical follow-up. The subsequent presentation with acute ischemic stroke prompted a thorough multidisciplinary investigation, which ultimately led to the diagnosis of cardiac involvement and systemic AL amyloidosis. Although atrial fibrillation is a well-recognized cause of cardioembolic stroke, patients with cardiac amyloidosis are at increased risk of intracardiac thrombus formation even in sinus rhythm because of atrial mechanical dysfunction and amyloid infiltration of the atrial myocardium [3]. Therefore, cardiac amyloidosis should be considered in the differential diagnosis of cryptogenic stroke, particularly when accompanied by findings suggestive of an infiltrative cardiomyopathy [4].

Several clinical "red flags" were present in this case. The coexistence of nephrotic syndrome, low QRS voltage on electrocardiography, and increased ventricular wall thickness on echocardiography strongly suggested an infiltrative myocardial disease rather than true hypertrophy [5]. This apparent discrepancy between electrocardiographic voltage and myocardial thickness is a classic feature of cardiac amyloidosis and should prompt further diagnostic evaluation [5-7].

CMR played a crucial role in establishing the diagnosis. The markedly elevated native T1 values, substantially increased ECV, and diffuse non-ischemic LGE involving both the ventricles and atria were highly characteristic of cardiac amyloidosis. CMR has emerged as one of the most valuable noninvasive imaging modalities for the evaluation of infiltrative cardiomyopathies, particularly when echocardiographic findings are suggestive but not diagnostic [6].

Interestingly, serum and urine immunofixation studies and bone marrow biopsy were inconclusive despite the presence of systemic AL amyloidosis. The diagnosis was ultimately confirmed by renal biopsy demonstrating monoclonal lambda light-chain deposition. This finding highlights that negative hematological investigations do not completely exclude AL amyloidosis and that tissue biopsy from an affected organ remains the diagnostic gold standard when clinical suspicion persists [6-9].

The patient received daratumumab-based chemotherapy (Dara-CyBorD), which is currently considered the preferred first-line treatment for newly diagnosed AL amyloidosis. Nevertheless, despite appropriate therapy, she experienced rapid clinical deterioration with progressive renal failure and multiorgan failure, ultimately resulting in death. These all suggest the aggressive natural history of advanced AL cardiac amyloidosis and emphasize the importance of recognizing the disease before irreversible organ damage has occurred [10].

This case underscores the diagnostic value of integrating clinical findings, electrocardiography, multimodality cardiac imaging, and tissue histopathology in patients with suspected cardiac amyloidosis. Early identification of characteristic clinical and imaging features may facilitate timely diagnosis and treatment, potentially improving outcomes in this rapidly progressive disease [9].

## Conclusions

This case highlights the importance of maintaining a high index of suspicion for cardiac amyloidosis in patients presenting with unexplained stroke, nephrotic syndrome, increased ventricular wall thickness, and low QRS voltage on electrocardiography. Multimodality imaging, particularly CMR, played a pivotal role in raising diagnostic suspicion when conventional laboratory investigations were inconclusive. Histopathological confirmation by renal biopsy ultimately established the diagnosis of AL amyloidosis. Despite prompt diagnosis and initiation of daratumumab-based chemotherapy, the patient's rapidly progressive course underscores the aggressive nature and poor prognosis of advanced AL cardiac amyloidosis. Early recognition through careful integration of clinical, imaging, and pathological findings remains essential to improve patient outcomes.
